# High-Voltage Injuries and Train Surfing: A 30-Year Review of Epidemiology, Treatment, and Outcomes

**DOI:** 10.3390/jcm14092918

**Published:** 2025-04-23

**Authors:** Viktoria Koenig, David Lumenta, Julian Joestl, Gerald Ihra, Marita Windpassinger, Maximilian Monai, Alexandra Fochtmann

**Affiliations:** 1 Division of Plastic, Aesthetic and Reconstructive Surgery, Medical University of Vienna, Waehringer Guertel 18-20, 1090 Vienna, Austria; n11927646@students.meduniwien.ac.at (M.M.); alexandra.fochtmann@meduniwien.ac.at (A.F.); 2 Division of Plastic, Aesthetic and Reconstructive Surgery, Medical University Graz, Neue Stiftingtalstraße 6, 8010 Graz, Austria; david.lumenta@gmail.com; 3 Private Clinic—Priv.-Doz. Dr. Julian Joestl, PhD, MSc. Spitalgasse 19, 1090 Vienna, Austria; 4 Division of General Anaesthesia and Intensive Care Medicine, Medical University of Vienna, Waehringer Guertel 18-20, 1090 Vienna, Austria; gerald.ihra@meduniwien.ac.at (G.I.);

**Keywords:** high-voltage electrical injuries, train surfing, burns, reconstruction, burn injuries, electrical arcs, train climbing, electrical injury

## Abstract

**Background:** High-voltage injuries associated with train surfing are a distinct subset of electrical injuries, yet detailed analyses remain limited. This study retrospectively reviewed train-surfing injuries admitted between 1994 and 2024, comparing their characteristics and outcomes to work-related high-voltage injuries. **Methods:** Medical records of 102 patients admitted for high-voltage injuries were analyzed, including 32 train-surfing and 70 work-related cases. Demographics, injury patterns, and clinical outcomes were assessed. **Results:** Train surfers were predominantly young males (median age 19 years), while work-related injuries involved slightly older males (median age 34 years). Train surfers sustained more severe burns (%TBSA: 47.6% vs. 25.4%, *p* < 0.0001) and higher ABSI scores (6.7 vs. 5.3, *p* < 0.01). Vertical electrical flow was predominant in train surfing (65.6%), reflecting contact with overhead lines, while work-related injuries showed varied flow patterns, with diagonal flow being most frequent (58.6%). Train surfers had longer ICU stays (38.7 vs. 17.9 days, *p* < 0.001) and underwent more surgeries per patient (5.3 vs. 2.8, *p* < 0.01). Fasciotomy rates were significantly higher among train surfers (84.4% vs. 55.7%, *p* < 0.01), as were amputations (53.1% vs. 25.7%, *p* < 0.001). Mortality rates were similar in both groups (25%). **Conclusions:** Train-surfing injuries represent a distinct and highly severe subgroup of high-voltage trauma, marked by greater burn extent, predominantly vertical electrical flow due to contact with overhead lines, and significantly higher surgical complexity—including increased rates of fasciotomies and amputations. Despite comparable mortality, the clinical burden for train-surfing victims is substantially higher, reflected in longer ICU stays and more operations per patient. These findings underscore the urgent need for targeted prevention strategies addressing youth engagement in train surfing. Public health campaigns, railway infrastructure modifications (e.g., deterrent systems or physical barriers), and early educational interventions could play a critical role in reducing these preventable injuries. Furthermore, trauma centers should be prepared for the specific reconstructive and critical care demands posed by this high-risk group, emphasizing the importance of specialized multidisciplinary management protocols.

## 1. Introduction

High-voltage electrical injuries present a complex challenge in trauma and reconstructive surgery, often affecting multiple organ systems [1,2]. These injuries predominantly impact young men, with a reported 90% male predominance over recent decades [3]. Among them, train surfing—a high-risk behavior involving riding on the exterior of moving trains—has emerged as a significant and growing cause of high-voltage electrical injuries, particularly among adolescent and young adult males. This activity is often driven by thrill-seeking behavior or socio-economic pressures and is increasingly propagated through social media, where it is portrayed as a form of daring performance. A clear distinction must be made between train surfing, which involves riding on moving trains, and train climbing, which refers to scaling stationary train carriages [4]. In both scenarios, individuals are exposed to extreme danger by entering the vicinity of overhead railway power lines, which can carry up to 15,000 volts. Importantly, electrocution may occur even in the absence of direct contact, as high-voltage arcs can discharge through the air and cause devastating injuries. In addition to deep electrical burns and life-threatening systemic complications such as arrhythmias or rhabdomyolysis-induced renal failure, many patients sustain severe mechanical trauma, including skull fractures, spinal injuries, and polytrauma resulting from high-velocity falls or collisions with infrastructure [2,3,5,6,7]. Despite its rising incidence, train surfing remains understudied relative to other high-voltage injury mechanisms, highlighting a critical gap in trauma and burn care literature [8].

Train surfing injuries result from high-voltage contact with overhead power lines or exposure to electrical arcs. These arcs, capable of generating temperatures between 3000 and 20,000 °C, cause severe thermal and electrical trauma, including deep third-degree burns and extensive tissue damage. Falls from trains often exacerbate the injuries, leading to traumatic brain injuries, fractures, and spinal cord damage [9,10,11].

Due to their multisystem nature, high-voltage injuries demand a multidisciplinary approach [6,12,13]. Common treatments include decompression to prevent compartment syndrome, amputations, and complex soft-tissue reconstruction after extensive debridement [14]. Vascular damage caused by electrical currents often results in progressive tissue necrosis, complicating wound management. Microsurgical techniques are crucial, though the timing of reconstruction is debated: early reconstruction risks incomplete debridement, while delays increase infection risks [5,10,15].

Train surfing injuries differ significantly from occupational high-voltage injuries in patient demographics and patterns [8]. Occupational injuries typically involve men in their thirties and are less severe, while train surfing often results in more extensive trauma among younger individuals [6,10].

The authors revisited this topic after a noticeable increase in train surfing incidents in 2021 [4]. This rise in accidents—particularly among younger individuals—prompted the initiation of an awareness and prevention program in collaboration with schools and the national railway services. As a result, this served as a trigger for conducting a retrospective data analysis focusing on high-voltage electrical injuries treated in our Level I ICU burn center. An increasing number of such injuries, often affecting adolescents and young adults involved in train surfing or climbing on railway infrastructure, was observed [4].

This study provides a 30-year overview of high-voltage injuries at a single center, focusing on train-surfing-related cases. By analyzing patient demographics, injury mechanisms, and treatment outcomes, it aims to address gaps in the literature. The study compares train surfing injuries with other high-voltage trauma cases, examining decompression and amputation rates, soft-tissue coverage strategies, and complications, offering insights to optimize care and outcomes.

## 2. Materials and Methods

After ethical board approval (Ethics Committee approval number: 1384/2023), patient records from January 1994 to December 2024 were reviewed, focusing on individuals admitted with high-voltage injuries associated with train surfing. All data were extracted from our medical records data system.

The inclusion criteria encompassed all patients with documented high-voltage electrical injuries related to train surfing or due to work-related injuries. There were no restrictions regarding age or gender, as all patients affected by these rare types of injuries were included in the study. Patients with low-voltage injuries (<1000 V) or treated outside the department were excluded. A total of 102 patients were included in this study, ranging in age from 13 to 59 years. Of all patients, 5 were female.

The following parameters were analyzed: age, sex, circumstances of injury, total burn surface area (%TBSA), type of current flow through the body, associated injuries, length of ICU stay, number and type of surgical interventions (e.g., fasciotomy, amputation, defect coverage), and mortality rate.

Treatment protocols adhered to a standardized regimen, emphasizing fluid resuscitation with adjustments based on urinary output, hematocrit, and serum lactate levels. Myoglobinuria was managed using diuretics to maintain urinary pH, supported by central lines and hemodynamic and respiratory monitoring.

In line with updated standards, treatment has transitioned to a more advanced and individualized approach. Fluid resuscitation employs balanced solutions, such as crystalloids, albumin, and high-dose vitamin C, administered in minimal effective volumes. Dynamic monitoring through tools like transpulmonary thermodilution and PiCCO (pulse index contour continuous cardiac output), hemoglobin measurements, and echocardiographic assessments guides therapy. Circulatory support includes catecholamine therapy (e.g., noradrenaline, dobutamine), complemented by comprehensive ICU care encompassing oxygen therapy, thermoregulation, and analgesia to ensure precise and tailored patient management. Initial diagnostic work-up included ENT and ophthalmology consultations, ultrasound, and CT scans for stabilized patients. Blood tests, including creatine kinase and myoglobin levels, were monitored every 6 h initially and daily after the first 24 h.

Decompression surgeries, including fasciotomies and carpal tunnel release, were performed within 24 h for circular burns, entry/exit wounds, or other significant injuries. Elevated serum myoglobin or creatine kinase levels supported the surgical decision-making process. Temporary polyurethane dressings were applied postoperatively, with early debridement and amputations performed as necessary. Burn wounds were disinfected and dressed daily. Full-thickness burns were debrided and covered with skin grafts, while free flaps were employed for defects involving vital structures or when local soft tissue resources were inadequate. Most patients undergoing microsurgical reconstruction underwent preoperative CT angiography to evaluate recipient vessels.

### 2.1. Statistics

Statistical analysis was undertaken using R Version 3.1.1 SPSS (Armonk, New York, NY, USA). Continuous variables were analyzed using the Student’s *t*-test for parametric data and the Mann–Whitney U test for non-parametric data. Categorical variables were assessed using the chi-square test or Fisher’s exact test, where appropriate. Correlations between current flow type and complications such as cardiac failure and renal dysfunction were evaluated using contingency tables and chi-square tests. Results were considered statistically significant at *p* < 0.05. Parametric data are reported as mean ± standard deviation, while non-parametric data are presented as median [minimum–maximum].

### 2.2. Declaration of Generative AI and AI-Assisted Technologies

During the preparation process of this work, ChatGPT (version: GPT-4) was used in order to visualize the analyzed data. After using this tool, the authors reviewed and edited the content as needed and take full responsibility for the content of the publication.

## 3. Results

This study analyzed a total of 102 patients divided into two distinct categories: train-surfing or -climbing injuries (32 cases) and work-related high-voltage injuries (70 cases).

Train surfers were predominantly young males, with 97% of cases involving males and a median age of 19 years (range: 13–36 years). In contrast, work-related injuries were more common among slightly older individuals, with a median age of 34 years (range: 18–59 years) and 94% of cases involving males. The difference in gender distribution was not statistically significant (*p* = 0.9459), while the difference in age was highly significant (*p* < 0.0001) (Table 1).

The extent of injuries differed significantly between the two groups. Train surfers sustained more severe burns, with an average total body surface area (TBSA) of 47.6% (±20.1%), a median of 50%, and a range from 5% to 80%. Meanwhile, work-related injuries exhibited a lower average TBSA of 25.4% (±17.8%), with a median of 22% and the same range.

**Figure 1:**
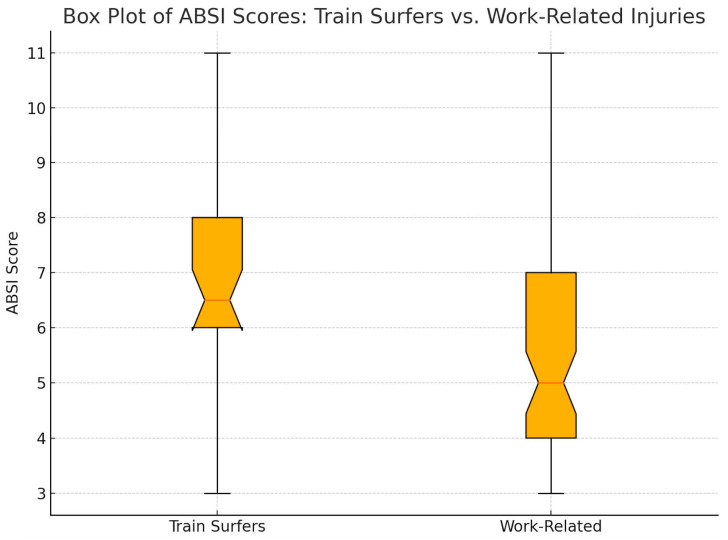
ABSI scores of train surfers and work-related injuries.

The analysis of Abbreviated Burn Severity Index (ABSI) scores reveals notable differences between train-surfing injuries and work-related high-voltage injuries. Train surfers exhibited a mean ABSI score of 6.7, with scores ranging from 3 to 11. In comparison, work-related cases had a lower mean ABSI score of 5.3, with the same range of 3 to 11 (*p* < 0.001) (Figure 1).

**Figure 2:**
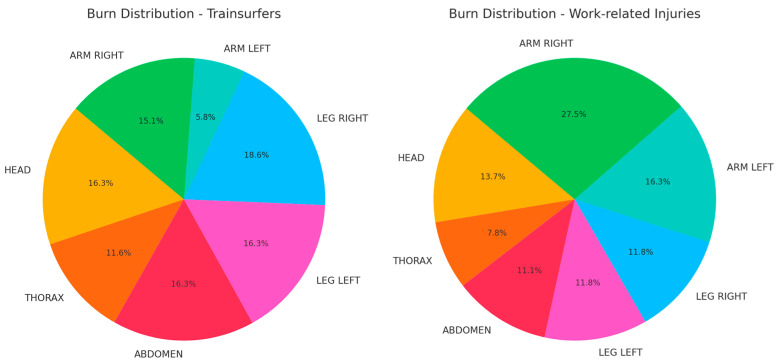
Burn distribution areas in train surfers and work-related injuries.

Burn distribution by body region differed between the two groups. Among train surfers, the most frequently affected areas were the right leg (50.0%), head (43.8%), left leg (43.8%), and right arm (40.6%). Thoracic burns were present in 21.9%, while abdominal involvement was observed in 18.8% of cases. In work-related injuries, the right arm was the most commonly affected region (60%), followed by the left arm (35.7%), head (30.0%), and both legs (25.7% each). Thoracic and abdominal burns occurred in 20% and 15.7% of work-related cases, respectively. Statistical analysis using chi-squared tests revealed a significant difference in the frequency of right leg burns between the groups (*p* = 0.0287), with these injuries being more prevalent among train surfers. Other regions, including the upper limbs and head, showed trends toward group differences, but these did not reach statistical significance (Figure 2).

**Figure 3:**
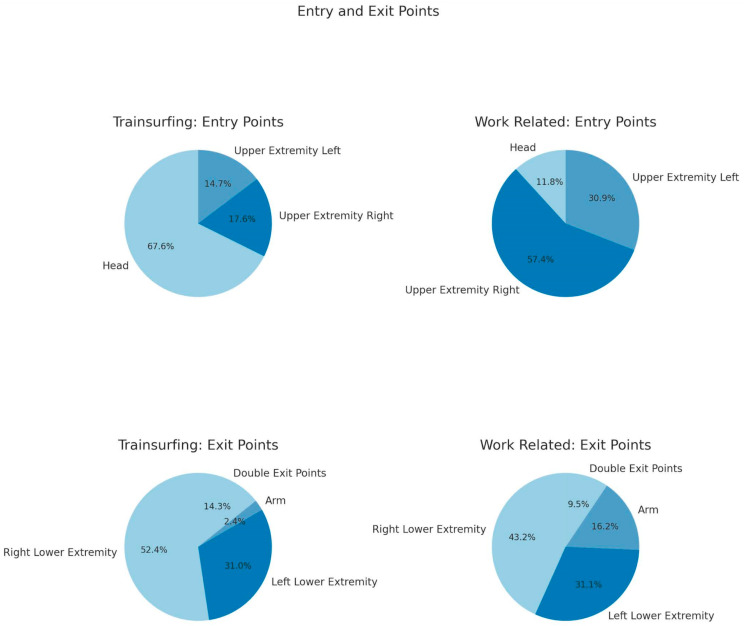
Entry and exit points.

Analysis of electrical current flow patterns revealed distinct differences between the two groups. For train-surfing patients, entry points most frequently involved the head and upper extremities, while exit points were predominantly recorded in the lower extremities and the arm. In contrast, work-related incidents showed a higher frequency of injuries to the upper extremities (both right and left) at entry points, with exit points primarily affecting the lower extremities and arms (Figure 3).

In train-surfing incidents, vertical current flow dominated, occurring in 65.6% of cases, and led to more cardiac complications such as arrhythmia in 37.5% of cases. This pattern reflects frequent direct contact with high-voltage power lines from above. Horizontal flow was nearly absent, while diagonal flow occurred in 21.9% of cases. Conversely, work-related incidents demonstrated a greater variety of current flows, with diagonal flow being most common (58.6%), followed by horizontal (10%) and vertical flows (7.1%). The difference in current flow distribution between the two groups was statistically significant (*p* < 0.0001).

The severity of injuries was further reflected in the clinical outcomes. Train-surfing patients had longer intensive care unit (ICU) stays, averaging 38.7 days (range: 1–270 days), compared to 17.9 days (range: 1–111 days) for work-related injuries.

Among the 32 train-surfing patients, 27 underwent fasciotomies, corresponding to a rate of 84.4%. In contrast, fasciotomies were performed in 39 of the 70 work-related injury patients, resulting in a lower rate of 55.7%. This difference in fasciotomy frequency was statistically significant (*p* = 0.0097).

Other surgical interventions highlighted the complexity of both categories, with a total of 365 surgeries performed across all patients. Train-surfing cases required more extensive interventions, averaging 5.3 surgeries per patient compared to 2.8 in the work-related group. Split-thickness skin grafts were performed in 23 of 32 train-surfing cases (71.9%) and in 48 of 70 work-related injuries (68.6%) (*p* = 0.0405).

Among all patients, 18 (17.6%) underwent local flap reconstruction, and 22 (21.6%) received a free flap. Flap surgeries were often required to cover exposed bone or vital structures, particularly in the scalp and lower extremity regions. The most frequently used free flaps were the M. latissimus dorsi flap (LD), anterolateral thigh flap (ALT), and M. gracilis flap.

In the train-surfing group (n = 32), 10 patients (31.3%) underwent local flap reconstruction, and 12 (37.5%) required a free flap. In the work-related injury group (n = 70), only eight patients (11.4%) underwent local flap procedures, and 10 (14.3%) received free flaps.

Statistical analysis showed that local flap reconstructions were significantly more frequent among train-surfing patients (*p* = 0.0310). While free flap procedures were also more common in this group, the difference did not reach statistical significance (*p* = 0.0683). Notably, some patients required multiple free flaps for adequate coverage, especially in cases of extensive scalp or lower limb injuries following high-voltage contact. A total of 4 of the 22 free flaps underwent revision, leading to three total flap losses, two of them seen in the train-surfing cohort.

Amputations were also notably more frequent among train surfers. A total of 17 train-surfing patients (53.1%) required amputations, compared to 18 patients (25.7%) in the work-related group. Among these train surfers who needed amputations, eight were macroamputations, representing 25.0% of the total cases. In contrast, for work-related incidents, seven were macroamputations, making up **10%** of the total cases. Amputations of the toes were required in five train-surfing patients (15.6%) compared to seven patients (10%) in the work-related group (*p* = 0.6263). Forefoot amputations were performed in four train surfers (12.5%) and two work-related patients (2.9%) (*p* = 0.1424). Lower leg (transtibial) amputations were exclusively observed in the train-surfing group, affecting four patients (12.5%), and none in the work-related group (*p* = 0.0136). Amputations of the fingers were required in two train surfers (6.2%) and three patients (4.3%) with work-related injuries (*p* = 1.0000). Transradial amputations were observed in two train-surfing cases (6.2%) and four work-related cases (5.7%). Transhumeral amputations were needed in one train-surfing patient (3.1%) versus three patients (4.3%) in the work-related group. A shoulder disarticulation (3.1%) occurred in one train-surfing case; no such case was documented in the work-related group (*p* = 0.6866).

Notably, two train-surfing patients required bilateral transtibial amputations, highlighting the extent of tissue destruction often associated with high-voltage contact involving both lower extremities.

Most amputations were observed at the site of electrical exit wounds, commonly affecting the toes, but also in patients with third-degree burns to the hands, particularly involving the fingers.

Transradial and transhumeral amputations, as well as one shoulder exarticulation, were seen both in work-related incidents, where patients were holding metallic tools or wires, and among train surfers who were handling graffiti spray cans at the time of high-voltage contact, resulting in current entry through the upper extremity, resulting in massive muscle damage and therefore amputation indication.

Train surfers were significantly more likely to require resuscitation (43.8% vs. 15.7%, *p* = 0.005), reflecting greater injury severity, though no differences in ECG abnormalities were observed (*p* = 0.325). Kidney failure affected 28.43% of patients, with higher rates in train surfers (43.8%) compared to work-related cases (21.4%), but no significant link was found between current flow type and kidney failure (*p* = 0.279).

Among all patients, 37 patients had associated traumatic injuries, representing 36.3% of all patients. Specifically, 18 train surfing patients had associated injuries, accounting for 56.3% of all train surfing patients. Additionally, 19 work-related patients had traumatic injuries, which corresponds to 27.1%. This difference was statistically significant (*p* = 0.0089).

**Figure 4:**
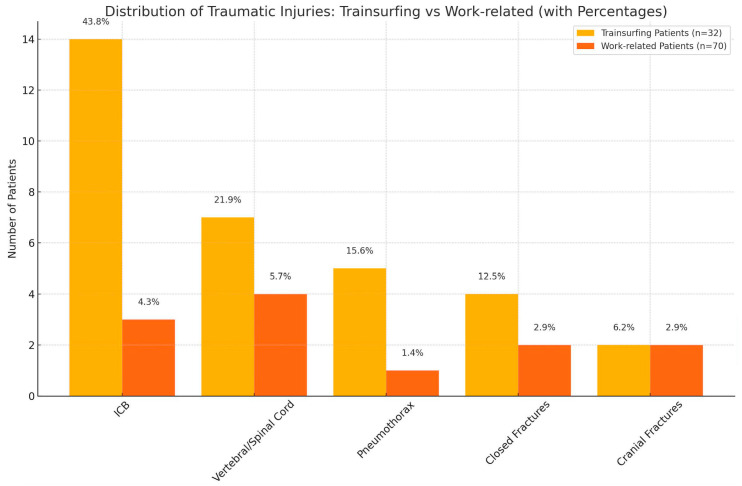
Traumatic injuries.

For all patients with associated traumatic injuries, the distribution of traumatic injuries is as follows: 13 patients had intracerebral bleeding (ICB); 8 had vertebral fractures or spinal cord injuries; 5 had pneumothorax; 3 had a combination of cranial fracture, ICB, and spinal cord injury; 2 had fractures of the upper limb; 2 had intracerebral bleeding, pneumothorax, and serial rib fractures; 2 had cranial fractures; 1 had a combination of cranial fracture, ICB, spinal cord injury, and fractures of the upper limb; and 1 had rib fractures (Figure 4).

Among train surfing patients with associated injuries, 14 experienced intracerebral bleeding (ICB), 7 suffered vertebral fractures or spinal cord injuries, 5 had pneumothorax, 4 sustained closed fractures, and 2 experienced cranial fractures.

For work-related patients with associated injuries, four suffered vertebral fractures or spinal cord injuries, three experienced intracerebral bleeding (ICB), two sustained closed fractures, two had cranial fractures, and one experienced pneumothorax.

Train-surfing incidents are associated with significantly elevated biomarker levels compared to work-related high-voltage injuries, reflecting the greater physiological burden of these traumas. To ensure comparability despite varying hospital stays, heart enzyme analysis was limited to the first 10 days after injury. Troponin measurements were mostly confined to the initial 48 h, focusing on acute myocardial injury. For patients hospitalized fewer than 10 days, all available data were included. Multiple daily measurements were averaged to calculate daily means. For example, average creatine kinase (CK) levels reached 20,778.73 U/L in train surfers versus 4097.50 U/L in work-related cases (*p* < 0.001), and mean myoglobin concentrations were 15,951.83 ng/mL compared to 3232.10 ng/mL, respectively (*p* < 0.001). These substantial differences indicate extensive muscle damage and rapid tissue breakdown in the train surfing cohort. This pattern is further supported by significantly higher cardiac-specific markers, such as Troponin, which averaged 309.08 ng/L in train surfers versus 27.91 ng/L in work-related injuries (*p* = 0.002).

Lactate dehydrogenase (LDH) levels were significantly higher in the train-surfing group across all measured values. The maximum LDH levels reached 3352 U/L compared to 2221 U/L in the work-related group (*p* < 0.001). Similarly, the mean LDH was 527.78 U/L versus 241.33 U/L, and the median was 365 U/L versus 177 U/L, respectively (all *p* < 0.001). These differences remained statistically significant after correction (*p* = 0.042).

Acute kidney failure was observed in 29 patients (28.4%), affecting 43.75% of train surfers and 21.4% of work-related cases (*p* = 0.031). Dialysis was required in 22 patients (21.6%), with a markedly higher incidence among train surfers (34.4%) compared to occupational injuries (15.7%) (*p* = 0.022). However, no statistically significant correlation was found between the type of electrical current flow and the development of kidney failure (chi-square = 3.84; *p* = 0.279) (Table 2).

The analysis of mortality rates between work-related incidents and train-surfing cases reveals notable differences. Out of 70 documented work-related cases, 8 resulted in fatalities, leading to a mortality rate of 11.4%. In comparison, train-surfing accounted for 32 cases, also with 8 fatalities, yielding a considerably higher mortality rate of 25%. However, this difference did not reach statistical significance (*p* = 0.1456).

A multivariate logistic regression analysis was performed to identify predictors of mortality among 102 patients with high-voltage injuries. After adjusting for confounders, TBSA (OR 1.08, 95% CI 1.02–1.15, *p* = 0.009), resuscitation (OR 4.56, 95% CI 1.11–18.79, *p* = 0.035), and the presence of acute kidney failure (OR 3.27, 95% CI 1.09–9.76, *p* = 0.034) were independently associated with mortality. Membership in the train-surfing group showed a trend toward higher mortality but did not reach statistical significance (OR 2.11, 95% CI 0.79–5.63, *p* = 0.129).

## 4. Discussion

This study provides a comprehensive analysis of train-surfing and work-related high-voltage injuries, offering new insights into demographic trends, injury patterns, and clinical outcomes. The differences between the two groups underscore the diverse mechanisms and consequences of these injuries.

Train-surfing injuries predominantly affected young males with a median age of 19 years, while work-related injuries were more common among slightly older individuals, with a median age of 34 years. This demographic pattern reflects the distinct nature of these incidents, with train-surfing linked to high-risk, thrill-seeking behavior and occupational injuries occurring in structured professional environments. These findings are consistent with Lumenta et al. and Maghsoudi et al., who also identified younger males as the primary victims of non-occupational high-voltage injuries [10,15]. Similarly, Butler et al. reported an average age of 36 years among linemen involved in professional electrical accidents [7]. These data are also consistent with previous findings by Lin et al., who noted that risky behaviors, such as train surfing, often attract adolescents and young adults seeking thrills [16]. In contrast, work-related injuries typically affect professionals exposed to high-voltage environments, as reported by Hussmann et al., where occupational injuries were predominantly seen in older individuals [2]. These findings emphasize the need for tailored preventive strategies for both at-risk groups [17].

When compared to recent published data from the Helsinki Burn Centre, our cohort reveals similar demographic and injury patterns among patients sustaining high-voltage injuries due to train surfing or climbing [8]. Both studies report predominantly male adolescents as the affected population, with median ages of 15.5 years in the Helsinki cohort and 19 years in our study. The average TBSA was comparable (45% vs. 47.6%), and both cohorts required a high number of surgical interventions during prolonged hospital stays. However, our cohort showed a higher in-hospital mortality rate (25.0% vs. 16.7%) and a greater incidence of major amputations (25% vs. 16.7%). Unlike the Helsinki study, we included a control group of work-related high-voltage injuries and performed a multivariate logistic regression to identify independent predictors of mortality.

Over the past decades, train surfing has remained a highly dangerous activity, with social media now fueling its resurgence as a global trend. Various platforms not only glorify the activity but even provide detailed route information and tips on where and how to engage in train surfing or train climbing. This widespread digital influence has contributed to an increase in participants, often young individuals seeking online recognition, thereby exacerbating the risks and frequency of related injuries, especially in young male adolescents [8,18,19].

Train surfers typically access train roofs through windows or unlocked doors, often transitioning from one side of the train to the other. While climbing, they maintain a grip on supporting structures but frequently release their hold while standing on top of moving carriages, exposing themselves to uncontrolled movement and extreme instability. This behavior explains why, in our study cohort, injuries affected nearly all body regions [5,10,18].

A particularly striking observation in our cohort was the higher incidence of associated traumatic injuries. We hypothesize that the continuous motion and speed of trains significantly increase the risk of secondary trauma, as individuals are often forcibly thrown off the train upon losing balance or contact with the carriage. Additionally, train surfers possibly stand at a greater height on the roof of the train, whereas occupational accidents involving high-voltage injuries typically occur at ground level during track maintenance work, resulting in different injury mechanisms and impact forces [5,16]. This fact significantly increases the risk of secondary trauma, as individuals are often forcibly thrown off the train upon losing balance or contact with the carriage. While the shorter contact time with the electrical source may reduce the severity of direct electrical burns, the resulting mechanical trauma is significantly more severe in cases of survival [20,21].

Our patient cohort, similar to Lumenta’s study, exhibited a notably higher rate of associated injuries, further supporting the assumption that train surfers are frequently ejected from the train, leading to severe traumatic injuries such as head trauma, fractures, and spinal injuries [10]. Unfortunately, literature on this specific topic remains scarce, with most data on associated injuries among train surfers stemming from forensic reports rather than clinical studies. Post-mortem analyses align with our findings, with train surfing fatalities most commonly involving the brain, heart, spine, and eyes [16,22,23]. This highlights an urgent need for more clinical research on non-fatal cases, as well as enhanced public awareness and prevention efforts to mitigate the growing risks associated with this dangerous social media-driven trend.

The patterns of injuries further highlight the contrast between these two groups. Train-surfing incidents resulted in significantly greater TBSA burns, with a median of 50% compared to 22% in work-related injuries. This is probably due to the closer high-energy contact with overhead power lines, leading to predominantly vertical current flow in 65.62% of cases. This aligns with Zhu et al.’s observation that severe burns and deep tissue damage are prevalent in incidents involving near contact to overhead wires [24].

The mechanism of current flow potentially plays a crucial role in determining the extent of muscle damage and internal organ involvement, particularly cardiac complications. In train surfers, vertical current flow is predominant, often running from the head or upper extremities through the lower body, which has been strongly associated with cardiac arrest and severe internal injuries. This differs significantly from work-related high-voltage injuries, where diagonal or transverse current flow is more common due to the posture and positioning of workers during track maintenance. These variations in electrical current pathways likely result in distinct patterns of internal injuries, with train surfers potentially experiencing more severe cardiac and neurological damage, while occupational injuries may involve greater musculoskeletal and soft tissue trauma due to possible prolonged current exposure [20,21,25,26].

Our findings are consistent with Lumenta et al., who reported a high prevalence of vertical current flow (83%) in train-surfing accidents, aligning with similar observations by Ofer et al. in severe electrical injuries [10,27]. Maghsoudi et al. further emphasized the correlation between high-voltage contact and extensive burns, supporting the notion that current flow patterns strongly influence injury severity [15]. Interestingly, while Lumenta et al. observed limited upper extremity involvement in train surfers, our study documented a higher incidence of upper limb injuries, potentially linked to modern behaviors such as graffiti spraying [10]. In contrast, work-related injuries showed a higher prevalence of diagonal current flow (58.57%), reinforcing the findings of Ferreiro et al., who highlighted the influence of current direction on injury distribution and severity [28].

The significantly higher fasciotomy rate among train-surfing patients (84.4%) compared to work-related high-voltage injuries (55.7%) underscores the severity of tissue damage and compartment syndrome risk in this patient population. Arnoldo et al. stated that train surfing involves uncontrolled high-energy trauma, which may contribute to a higher incidence of severe soft tissue and vascular injuries, necessitating urgent decompression. In contrast, work-related injuries often occur in a more controlled environment, where immediate medical intervention may mitigate the need for fasciotomies [3].

The complexity of surgical management in both groups is reflected in the total of 365 surgeries performed in our patents, with train-surfing patients undergoing an average of 5.3 surgeries per patient—almost twice the number required for work-related injuries (2.8 per patient). This highlights the greater reconstructive burden in train-surfing accidents. The higher frequency of flap surgeries (50% vs. 31.4%) further supports this observation, suggesting that train-surfing patients required more extensive soft tissue reconstruction to salvage viable limbs.

Notably, the amputation rate was significantly higher among train surfers (53.1%) compared to work-related injuries (25.7%). This difference can likely be attributed to the combination of high-voltage electrocution and severe secondary trauma sustained in train-surfing accidents, potentially leading to a delayed reconstruction. Patients suffering from intracranial bleeding due to falls or blunt trauma associated with train-surfing accidents may have been clinically unstable, delaying urgent limb-saving procedures. This highlights the complex interplay between polytrauma, neurotrauma, and electrical injuries, necessitating a multidisciplinary approach to optimize outcomes [29,30,31].

The high incidence of macroamputations (25% of train surfers vs. 10% of work-related cases) emphasizes the destructive nature of these injuries, where multiple limb segments are often non-salvageable due to extensive tissue necrosis, vascular damage, and secondary infection risk.

Our findings are consistent with Cancio et al., who identified full-thickness burns and myoglobinuria as key predictors for both fasciotomy and amputation, emphasizing the importance of early and aggressive decompression in high-voltage injuries [11]. In our cohort, all clinically indicated fasciotomies were performed within the first 24 h after admission, following the exclusion of other life-threatening injuries.

Over the decades, amputation rates for similar injuries have shown a marked decline, from 68% in the 1970s to 19% in 2009, likely due to advancements in early fasciotomy protocols, reconstructive techniques, and improved critical care management [11]. Cancio et al. underscored the crucial role of timely fasciotomy in mitigating further complications, while Butler et al. highlighted that amputations remain necessary in cases of extensive muscle necrosis or delayed intervention [7,11]. Furthermore Luce et al. emphasized the importance of aggressive wound management and timely surgical intervention [32].

Mann et al. reported that in a cohort of 100 upper extremity high-voltage injuries, the amputation rate was only 10% with a decompression rate of 22% per extremity, and none of the non-decompressed extremities required amputation [33]. This raises the question of overutilization of fasciotomy in some cases and whether a more refined selection process—potentially incorporating perfusion imaging or biomarkers of ischemic injury—could optimize decision making while minimizing unnecessary procedures.

Overall, our data suggest that train surfing injuries necessitate a more aggressive surgical approach due to their unique combination of electrical and traumatic injury patterns. While our early decompression strategy appears justified given the severity of injuries observed, further prospective studies are needed to establish more precise indications for fasciotomy, particularly in polytraumatized electrical injury patients. Additionally, preventive efforts, including public awareness campaigns, remain crucial to addressing the growing incidence of train-surfing accidents, which continue to impose a high surgical and socioeconomic burden.

The higher rate of flap surgeries among train-surfing patients in our study (50%) compared to work-related injuries (31.4%) highlights the significant reconstructive challenges associated with these injuries. This contrasts with Lumenta et al.’s findings, which reported lower rates of soft-tissue reconstruction in train surfers (25%), possibly reflecting differences in injury severity among patient cohorts [10]. The complexity of tissue damage in train-surfing accidents often necessitates multiple surgical procedures, including flap reconstruction, to achieve limb salvage and functional recovery, as emphasized by Maghsoudi et al. as well as in Korkiamakis study [8,15].

Koul et al. similarly highlighted the devastating nature of high-voltage injuries, which frequently result in extensive soft-tissue loss, limb loss, or even life-threatening conditions [34]. Deep structures are often exposed, requiring flap coverage, yet microvascular free flaps in electrical burns have traditionally been associated with higher failure rates. However, their study of 16 free tissue transfers in 13 patients demonstrated a high success rate, with only one flap failure due to vascular erosion and secondary hemorrhage, rather than vascular occlusion. Their findings suggest that with meticulous debridement and careful microvascular anastomosis away from the trauma zone, free flaps can achieve survival rates comparable to other indications, even in electrical burn patients.

Our findings further support the viability of reconstructive strategies, including free flap transfer, in managing severe electrical injuries. While train-surfing accidents often require more extensive reconstruction, the principles of early, well-planned microsurgical intervention remain crucial for optimizing outcomes [20,34].

Train surfers were significantly more likely to require resuscitation (43.8% vs. 15.7%), reflecting greater injury severity, though no differences in ECG abnormalities were observed (*p* = 0.325). Kidney failure affected 28.4% of patients, with higher rates in train surfers (43.8%) compared to work-related cases (21.4%), but no significant link was found between current flow type and kidney failure (*p* = 0.279).

While no immediate differences in ECG abnormalities were detected, it is plausible that cardiac complications in train-surfing injuries are influenced by the distinct current flow patterns observed in this group [35,36,37,38]. Vertical current flow, which is more common in train surfers, has been strongly associated with direct myocardial damage and fatal arrhythmias in our patients. However, further in-depth investigations are necessary to determine whether specific current flow patterns contribute to a higher risk of cardiac dysfunction. Additionally, future research should focus on potential long-term consequences of electrical injuries on the heart, including cardiomyopathy, conduction disorders, and structural damage, which remain poorly understood and rarely studied in this patient population.

The clinical outcomes further highlight the distinct nature of train-surfing injuries. Patients in this group experienced significantly longer ICU stays and a higher mortality rate (25%) compared to work-related cases (11.4%). This is consistent with findings from Sternick et al., who reported mortality rates of up to 44% in train-surfing injuries depending on access to care and injury complexity [5]. Lumenta et al. documented a lower mortality rate of 8%, which may be attributed to differences in sample size or healthcare advancements [10]. By contrast, work-related injuries benefited from structured environments and timely intervention, as noted by Maghsoudi et al. and Butler et al., who highlighted the role of safety protocols and rapid response in mitigating fatal outcomes [7,15].

Even Shih et al. stated in their review on Adult Electrical Burn Injury Outcomes that it becomes evident that most existing studies focus primarily on reporting general outcomes of electrical injuries. However, long-term complications—particularly regarding psychological impact and rehabilitation—are often underreported. This highlights significant gaps in the evaluation of key aspects of electrical injury [1].

These insights underline the critical role of early intervention and comprehensive surgical planning in optimizing outcomes for high-voltage injury patients. The comparison between train-surfing and work-related injuries emphasizes the need for tailored preventive measures, advanced reconstructive strategies, and improved healthcare accessibility to address the unique challenges posed by these injuries.

## 5. Conclusions

Over the past three decades, train surfing has become a high-risk activity with significant public health consequences. Nearly half of cases result in amputations, including macro amputations, and the activity is linked to high mortality and hospitalization rates, emphasizing its severe and life-altering impact.

Train surfing injuries differ markedly from work-related high-voltage incidents. Vertical current flow in train surfing causes severe burns and secondary trauma from falls, often leading to multi-system injuries like cranial bleeding and fractures. Our analysis suggests that vertical current flow in train surfing patients also poses a higher risk for cardiac failure.

These injuries demand prolonged ICU stays and carry a greater surgical burden. In contrast, work-related injuries, typically involving diagonal current flow, are more localized to the hands and arms due to tool use.

These distinct injury patterns require tailored prevention and treatment strategies. For train surfing, public awareness campaigns targeting young males and preventive measures such as barriers on trains are critical. Work-related injuries, meanwhile, call for stricter workplace safety protocols and enhanced training programs.

Given the increasing number of train surfing incidents associated with severe high-voltage injuries, the authors recognized the urgent need to inform relevant authorities and initiate targeted prevention efforts. Reaching the young population at risk, however, poses a significant challenge, as this group is often difficult to engage through traditional educational channels.

In response, an awareness project was launched in collaboration with the national railway authority. This ongoing, multimodal campaign includes print, online, and television media with a specific focus on educating adolescents and young adults about the life-threatening dangers of train surfing and climbing on railway infrastructure. Although the overall number of electrical injuries (EI) remains relatively low, the individual cases are highly traumatic—not only for the patients but also for their families.

To support affected individuals, a self-help group was established at our clinic. The project initially connected three male patients (aged 14, 17, and 19) and their families, who expressed great appreciation for the opportunity to share experiences and process the psychological impact of the trauma together.

Our findings underscore the critical need for early education about the risks of high-voltage injuries. Notably, none of the train-surfing patients treated in our center were aware of the dangers posed by electrical arcs before their accidents [4].

This awareness initiative reflects a proactive approach to injury prevention, emphasizing the importance of public education and outreach. By addressing both physical and psychosocial aspects of these injuries, the program aims to reduce the incidence of such preventable trauma and empower young individuals to make safer choices.

This 30-year analysis highlights the urgent need for focused interventions to reduce the human and systemic costs of these injuries and to improve outcomes through targeted safety strategies.

## 6. Limitations

As a retrospective analysis, the study is limited by potential inconsistencies in documentation and data availability over the 30-year period. Furthermore, due to the rarity of high-voltage injuries—particularly those related to train surfing—annual case numbers were low, which may reduce the statistical power and generalizability of subgroup comparisons.

## Figures and Tables

**Table 1:** Demographics: Train-Surfing vs. Work-Related Electrical Injury.

Variable	Train-Surfing	Work-Related
Total Patients	32	70
Male (%)	31/32 (97%)	66/70 (94%)
Female (%)	1/32 (3%)	4/70 (6%)
Median Age (years)	19	34
Age Range (years)	13–36	18–59

**Table 2:** Clinical and Surgical Comparison: Train-Surfing vs. Work-Related Electrical Injury.

Category	Train-Surfing	Work-Related	*p*-Value
TBSA Mean (%)	47.6 ± 20.1	25.4 ± 17.8	<0.001
TBSA Median (%)	50	22	<0.001
ABSI Score (Mean, Range)	6.69 (3–11)	5.31 (3–11)	
ICU Stay (days)	38.69 (1–270)	17.93 (1–111)	
Fasciotomy (%)	27/32 (84.38%)	39/70 (55.71%)	6
Surgeries per Patient (Mean, Range)	5.31 (1–24)	2.79 (1–14)	< 0.001
Split Skin Graft (%)	23/32 (71.88%)	48/70 (68.57%)	0.0405
Local Flap (%)	10/32 (31.3%)	8/70 (11.4%)	0.0310
Free Flap (%)	12/32 (37.5%)	10/70 (14.3%)	0.0683
Amputations Total (%)	17/32 (53.13%)	18/70 (25.71%)	5
Macroamputations (%)	8/32 (25.0%)	7/70 (10.0%)	43
Toe Amputations (%)	5/32 (15.6%)	7/70 (10.0%)	0.6263
Forefoot Amputations (%)	4/32 (12.5%)	2/70 (2.9%)	0.1424
Lower Leg Amputations (%)	4/32 (12.5%)	0/70 (0%)	0.0136
Resuscitation (%)	14/32 (43.75%)	11/70 (15.71%)	5
Kidney Failure (%)	14/32 (43.75%)	15/70 (21.43%)	31
Dialysis (%)	11/32 (34.38%)	11/70 (15.71%)	22
CK (U/L, Mean)	20,778.73	4097.50	<0.001
Myoglobin (ng/ml, Mean)	10,951.83	3232.10	<0.001
Troponin (ng/L, Mean)	309.08	27.91	2
LDH Mean (U/L)	527.78	241.33	<0.001
Mortality (%)	8/32 (25.0%)	8/70 (11.43%)	0.1456

## Data Availability

Data are available upon request.
